# Outcomes of Late-Line Systemic Treatment in GIST: Does Sequence Matter?

**DOI:** 10.3390/cancers16050904

**Published:** 2024-02-23

**Authors:** Prapassorn Thirasastr, Thomas L. Sutton, Cissimol P. Joseph, Heather Lin, Behrang Amini, Skye C. Mayo, Dejka Araujo, Robert S. Benjamin, Anthony P. Conley, John A. Livingston, Joseph Ludwig, Shreyaskumar Patel, Ravin Ratan, Vinod Ravi, Maria A. Zarzour, Elise F. Nassif Haddad, Michael S. Nakazawa, Xiao Zhou, Michael C. Heinrich, Neeta Somaiah

**Affiliations:** 1 Department of Sarcoma Medical Oncology, The University of Texas MD Anderson Cancer Center, Houston, TX 77030, USA; pthirasastr@mdanderson.org (P.T.); cpjoseph@mdanderson.org (C.P.J.); daraujo@mdanderson.org (D.A.); rbenjami@mdanderson.org (R.S.B.); aconley@mdanderson.org (A.P.C.); jalivingston@mdanderson.org (J.A.L.); jaludwig@mdanderson.org (J.L.); spatel@mdanderson.org (S.P.); rratan@mdanderson.org (R.R.); vravi@mdanderson.org (V.R.); maflores@mdanderson.org (M.A.Z.); efnassif@mdanderson.org (E.F.N.H.); msnakazawa@mdanderson.org (M.S.N.); xzhou@mdanderson.org (X.Z.); 2 Division of Surgical Oncology, OHSU Knight Cancer Institute, Oregon Health & Science University School of Medicine, Portland, OR 97239, USA; suttoth@ohsu.edu (T.L.S.);; 3 Department of Biostatistics, The University of Texas MD Anderson Cancer Center, Houston, TX 77030, USA; hyanlin@mdanderson.org; 4 Department of Musculoskeletal Imaging, Division of Diagnostic Imaging, The University of Texas MD Anderson Cancer Center, Houston, TX 77030, USA; bamini@mdanderson.org; 5 Cell and Developmental Biology, OHSU Knight Cancer Institute, Oregon Health & Science University School of Medicine, Portland, OR 97239, USA; heinrich@ohsu.edu

**Keywords:** gastrointestinal stomal tumor, ripretinib, avapritinib

## Abstract

**Simple Summary:**

Ripretinib, with reported broad activity against *KIT*-mutated gastrointestinal stomal tumors (GISTs), has been approved in the fourth-line setting, while avapritinib, which has robust activity against *PDGFRA* D842V and *KIT* activation loop (AL) mutations, has been approved for tumors with *PDGFRA* exon 18 mutations in any line of treatment. Avapritinib has fair activity against tumors with *KIT* exon 17 AL mutations or primary exon 9 mutations, as reported in the NAVIGATOR trial. That finding led to the National Comprehensive Cancer Network guidelines’ inclusion of avapritinib as an available option after progression of disease on other FDA-approved tyrosine kinase inhibitors. As ripretinib and avapritinib have shown overlapping activity against *KIT* secondary mutations, we investigated whether the benefit of trying one of these drugs remains after exposure to the other. This study reports no significant difference in efficacy of these drugs regarding the sequence in which they were administered to the patient.

**Abstract:**

Ripretinib and avapritinib have demonstrated activity in the late-line treatment of gastrointestinal stomal tumors (GISTs). We investigated whether patients previously treated with ripretinib benefit from avapritinib, and vice versa. Patients diagnosed with metastatic/unresectable GIST and treated with both drugs at two institutions in 2000–2021 were included. Patients were grouped by drug sequence: ripretinib–avapritinib (RA) or avapritinib–ripretinib (AR). Radiographic response was evaluated using RECIST 1.1. Kaplan–Meier and log-rank tests were used to compare time-to-progression (TTP) and overall survival (OS). Thirty-four patients (17 per group) were identified, with a median age of 48 years. The most common primary site was the small bowel (17/34, 50%), followed by the stomach (10/34, 29.4%). Baseline characteristics and tumor mutations were not significantly different between groups. Response rates (RRs) for ripretinib were 18% for RA and 12% for AR; RRs for avapritinib were 12% for AR and 18% for RA. Median TTPs for ripretinib were 3.65 months (95%CI 2–5.95) for RA and 4.73 months (1.87–15.84) for AR. Median TTPs for avapritinib were 5.39 months (2.86–18.99) for AR and 4.11 months (1.91–11.4) for RA. Median OS rates following RA or AR initiation were 29.63 (95%CI 13.8–50.53) and 33.7 (20.03–50.57) months, respectively. Both ripretinib and avapritinib were efficacious in the late-line treatment of GIST, with no evidence that efficacy depended on sequencing.

## 1. Introduction

The majority of gastrointestinal stromal tumors (GISTs) are driven by mutation in the KIT proto-oncogene, receptor tyrosine kinase (*KIT*), and platelet-derived growth factor receptor alpha (*PDGFRA*) genes, which are proto-oncogenes encoding the cell-surface receptors c-kit and PDGFRA protein in the type III receptor tyrosine kinase (RTK) family. These receptors are found on interstitial cells of Cajal in the gastrointestinal tract, resulting in a gain of function that leads to activation of the phosphatidylinositol 3-kinase (PI3K)–Akt pathway and unregulated cell proliferation [1,2,3]. For patients with unresectable or metastatic GIST, systemic treatment with tyrosine kinase inhibitors (TKIs) is the primary treatment. Imatinib is the established first-line therapy for GIST, with a reported RR of around 50–70% and progression-free survival (PFS) of around 20 months [4,5,6]. Sunitinib and regorafenib are approved for the second and third lines of treatment, respectively. In phase 3 studies, both therapies showed improved RR and PFS over placebo, but the extent of benefit was numerically smaller [7,8,9]; sunitinib demonstrated an RR of 6.8% and median PFS of 5.6 months, while regorafenib resulted in an RR of 4.5% and PFS of 4.8 months.

The heterogeneity of secondary *KIT* mutations after treatment with imatinib is one of the main reasons for the significantly lower efficiency of TKIs in later lines of therapy [10]. The advent of new drugs, with improved efficacy against previously resistant *KIT* mutations, allows for better growth inhibition and longer survival. The main locations of secondary mutations are the ATP-binding domain (exon 13 V654 (39.6%), exon 14 T670 (11.1%)) and the activation loop (AL; exon 17 (41%)), leading to drug-binding hindrance and a conformational shift to the active form [11]. Ripretinib is a tyrosine switch-control inhibitor that has dual mechanisms of targeting the switch pocket and activation loop and stabilizing the switch pocket in an inactive conformation [12]. Ripretinib has broad activity against secondary mutations resistant to imatinib, which include *KIT* exon 13 (V654A), exon 14 (T670I), and exon 17/18 (D816V, A829P) mutations. It was recently approved by the U.S. Food and Drug Administration (FDA) for fourth- and later-line treatment of advanced GIST, based on improvements in RR (11.8% vs. 0%), median PFS (6.3 vs. 1.0 months), hazard ratio (0.16, 95% confidence interval (95% CI) 0.10–0.27), and overall survival (OS, 18.2 vs. 6.3 months) as compared to the placebo [13].

Avapritinib is a type I TKI that can target the *PDGFRA* exon 18 D842V mutation that causes primary imatinib resistance. Avapritinib was approved by the FDA for any line of treatment for GIST with *PDGFRA* D842V and other exon 18 mutations, based on results from the phase 1 NAVIGATOR study, which showed an RR of 88% in patients with *PDGFRA* D842V [14]. Apart from activity against the *PDGFRA* D842V mutation, avapritinib also demonstrated robust activity against *KIT* D816V AL mutations [12,15,16]. A recent post hoc analysis evaluated the clinical efficacy of avapritinib in mutation subgroups of the NAVIGATOR study and the phase 1/2 CS3007-101 study [17,18]. Avapritinib had higher antitumor activity in patients with *KIT* AL^pos^ABP^neg^ (i.e., with AL mutation (*KIT* exon 17/18) but without the *KIT* ATP-binding pocket [ABP] mutation), with an RR of 31.4% (vs. 12.1% for the other patients), median PFS of 9.1 months (vs 3.4 months), and HR of 0.47 (95%CI 0.32–0.68, *p* < 0.0001) across all lines of treatment and an RR of 38.5% and PFS of 19.3 months in the second-line setting [18,19]. Additionally, in patients with *KIT* exon 9 mutations, the median PFS durations were 5.6 months and 3.7 months in the fourth-line and beyond-fourth-line settings, respectively. Based on this analysis, avapritinib has been used as a treatment option after disease progression during therapy with the standard FDA-approved TKIs for late-line GIST, especially for patients with documented *KIT* exon 17/18 and/or *KIT* exon 9 mutation, although it is not approved for this indication.

In preclinical models, most *KIT* exon 17 mutations are sensitive to both ripretinib and avapritinib, while avapritinib showed decreased activity for exon 13 V654A [12,20]. Ripretinib showed lower potency against the *KIT* D816V (exon 17) mutation as compared to avapritinib while exhibiting the greatest potency against the *KIT* exon 18 A829P mutation amongst the FDA-approved drugs for GIST [12]. As both avapritinib and ripretinib have shown somewhat overlapping activity against *KIT* secondary mutations, prior treatment with the first drug might lower the efficacy of the second drug or render it completely inactive. Thus, there could be an optimal sequence of administering these two drugs for treatment of late-line GIST. To date, no clinical study has sought to answer this important question, which has significant implications in treatment planning for patients with imatinib-resistant GIST. Hence, in this study, we sought to retrospectively analyze efficacy data for each drug, based on the sequence in which they were used, by comparing a ripretinib-before-avapritinib (RA) group with an avapritinib-before-ripretinib (AR) group.

## 2. Materials and Methods

### 2.1. Patient Selection

All patients with pathologically diagnosed advanced GIST who were treated with both ripretinib and avapritinib, regardless of lines of treatment and intervening therapy between the two drugs, at The University of Texas MD Anderson Cancer Center and OHSU Knight Cancer Institute were included in the study. The pathologic diagnoses were obtained by either biopsy or tumor resection and were confirmed by morphologic characteristics and immunohistochemical staining for markers, including KIT (CD117), by an expert sarcoma pathologist and mutation testing of the genes encoding KIT and PDGFRA receptor tyrosine kinases. The patients were identified using electronic medical record search engines (SlicerDicer search of the MD Anderson Epic database from January 2016 to December 2021 and search of the prospectively maintained OHSU Cancer Registry for GIST diagnoses between January 2000 and December 2021).

This study protocol was approved by the Institutional Review Boards of MD Anderson (Protocol 2021-0435) and OHSU (STUDY00020791).

### 2.2. Data Collection

Patients’ data were retrospectively reviewed from electronic medical records, and baseline characteristics, pathology results, mutation tests, and treatment details were collected uniformly into a Research Electronic Data Capture (REDCap) database at MD Anderson and a Microsoft Excel spreadsheet at OHSU that were housed on secure networks. The patients’ identifiers were confidentially collected and replaced by study numbers before data extraction for analysis. Informed consent was not required due to the study’s retrospective and non-interventional design.

### 2.3. Treatment

All patients’ treatment decisions were made by the treating physician as part of standard treatment or the clinical trial protocols in effect at the time. Ripretinib was approved by the FDA as standard treatment for fourth- or later-line treatment of GIST on 15 May 2020, and avapritinib was approved for patients with the *PDGFRA* D842V mutation on 9 January 2020. Prior to drug approval, most of the patients received these therapies on clinical trials (NCT02571036, NCT03673501, NCT03353753, or NCT04148092 for ripretinib and NCT02508532, NCT03465722, or NCT03862885 for avapritinib).

### 2.4. Efficacy Assessment

Treatment response was evaluated by computerized tomography (CT) scans at baseline before the start of treatment and every 2–3 months per standard clinical practice or as dictated by the clinical study protocol. Response (best response and time to progression (TTP) of disease) was evaluated using Response Evaluation Criteria in Solid Tumors (RECIST) 1.1 by an independent radiologist (B.A). Patients with either complete response or partial response (PR) were included in the response rate calculation. Longer-term outcomes were evaluated for TTP and OS. Subgroup analysis was performed based on mutational status.

### 2.5. Statistical Analyses

The patients were divided into two groups: those who received ripretinib before avapritinib (RA group) and those who received avapritinib before ripretinib (AR group). Descriptive statistics (frequency distribution, mean (±s.d.) and median (range)) were used to summarize patient characteristics. The chi-squared test or Fisher’s exact test was used to test differences between groups for categorical variables, and the Wilcoxon rank-sum test or Kruskal–Wallis test was used to detect differences for continuous variables [21]. The distributions of TTP and OS were estimated by the Kaplan–Meier method [22]. TTP was defined by treatment agent. Time to first progression on ripretinib was defined as the time from treatment initiation of ripretinib at 150 mg daily to the time of progression or the time of discontinuation of that dose. Time to second progression on ripretinib was defined as the time from treatment initiation of ripretinib at 300 mg (150 mg twice daily) to the time of progression or the time of discontinuation of that dose. Time to progression on avapritinib was defined as the time from treatment initiation of avapritinib to the time of progression or discontinuation of avapritinib.

OS was defined as the time from start of treatment with the first of the two drugs to either death or last follow-up time if a patient was alive at the time of data collection. The log-rank test [23] was performed to test the difference in survival between groups.

## 3. Results

### 3.1. Patients and Treatments

We identified 34 patients with pathologically confirmed metastatic or unresectable GIST who had received ripretinib and avapritinib: 20 patients from MD Anderson and 14 patients from OHSU. Of the 34 patients, 17 patients were treated with ripretinib before avapritinib (RA group) and 17 patients were treated with avapritinib before ripretinib (AR group).

For the 34 patients together, the median age at diagnosis was 48 years (range 20.3–76.3; Table 1). Most of the patients were male (22 patients, 64.7%). The most common primary site was the small intestine (17 patients, 50.0%), and most patients had a spindle cell histologic subtype (23 patients, 67.7%). KIT exon 11 mutation was the most common primary mutation (20 patients, 58.8%), followed by KIT exon 9 mutation (9 patients, 26.5%). The baseline characteristics, including the mutation profile, of patients did not differ significantly between the RA and AR groups. However, two different patients in the AR group had KIT exon 13 or exon 17 as their primary mutation. In addition, two patients had PDGFRA D842V in the RA group, while only one patient had this mutation in the AR group. Secondary mutations in KIT exons 13 and 17 were found in both groups. One of the patients in the RA group had a mutation in PDGFRA D842V detected on liquid biopsy at the time of progression on imatinib; this patient had a baseline primary KIT exon 11 mutation.

All of the gastric GISTs in our cohort had the KIT exon 11 primary mutation (*n* = 10), and subsequently, secondary KIT exon 13 and/or 17 mutations were found in three of these patients (Table S1, Supplementary Data). Of small-bowel GISTs, 52.9% (9/17) had the exon 9 primary mutation, while 41.2% (7/17) had exon 11 as the primary mutation. None of these tumors with the KIT exon 9 mutation demonstrated additional secondary mutations, while one of the tumors with the primary exon 11 mutation had a secondary KIT exon 17 mutation, and another case was found by liquid biopsy to have a secondary PDGFRA D842V mutation. All three cases with primary PDGFRA D842V mutations had intra-abdomen, not otherwise specified, as the tumor site.

At initial diagnosis, the disease was localized in 14 patients (41.2%), locally advanced in 11 patients (32.4%), and metastatic in 9 patients (26.5%). Most of the patients in the AR group presented with localized disease (nine patients, 52.9%), while more patients in the RA group presented with locally advanced (six patients, 35.3%) or metastatic disease (six patients, 35.3%), although these differences in percentages did not reach statistical significance. Nonetheless, at the start of ripretinib or avapritinib (whichever was first), all patients had documented metastatic disease.

Surgery was performed as a primary treatment in all of the cases with localized disease, with one patient in each group receiving additional neoadjuvant/adjuvant systemic treatment (1/5 in the RA group and 1/9 in the AR group). For cases with locally advanced disease, most patients received surgery and systemic treatment as their primary treatment (8/11, 72.7%), while one patient in the AR group did not achieve volumetric reduction in tumor size enough to undergo tumor resection after TKI treatment as planned. Although more patients in the RA group presented with metastatic disease, four of them (4/6) underwent surgical resection of their primary tumor, and three also had a metastatectomy. The two remaining patients in the RA group and all three patients with metastatic disease in the AR group received systemic treatment alone as their primary treatment. The details of the operations are summarized in Table S2 (Supplementary Data).

The median number of lines of treatment at the start of the first of the two drugs was five in both the RA and AR groups (Table 2). Most of the patients (23/34, 67.6%) went directly either from ripretinib to avapritinib in the RA group or from avapritinib to ripretinib in the AR group. One patient in the RA group received local treatment (resection of peritoneal metastases) twice during ripretinib treatment for responding disease and after progression of the disease. Conversely, three patients in the AR group received some form of local treatment during ripretinib treatment; one received yttrium-90 radioembolization for progressing disease in the liver, and the others received local treatment for responding disease. One patient in each group received local treatment during avapritinib treatment, for stable disease (SD) or responding disease in the RA patient and progression of disease in the AR patient. The median durations of treatment for ripretinib were 11.6 months (range 1.93–45) in the RA group and 7.13 months (range 0.3–49.3) in the AR group, while the median durations of treatment for avapritinib were 5.15 months (range 0.87–54.77) in the RA group and 3.67 months (range 0.1–65.13) in the AR group. More patients (11 patients, 64.7%) in the RA group received dose escalation of ripretinib from 150 mg daily to 300 mg daily (150 mg twice-daily dosing) after disease progression, as per the protocol in either the phase 1 dose-escalation phase [24] or as part of the INVICTUS phase 3 study [25], compared to only four patients (23.5%) in the AR group who received ripretinib dose escalation. The median durations of ripretinib treatment listed above included the total duration of both dosing schedules. Nonetheless, with regard to the treatment group (RA vs. AR), neither the treatment duration for ripretinib nor that for avapritinib significantly differed statistically (*p* = 0.26 and 0.67 for ripretinib and avapritinib, respectively).

### 3.2. Efficacy

**Figure 1:**
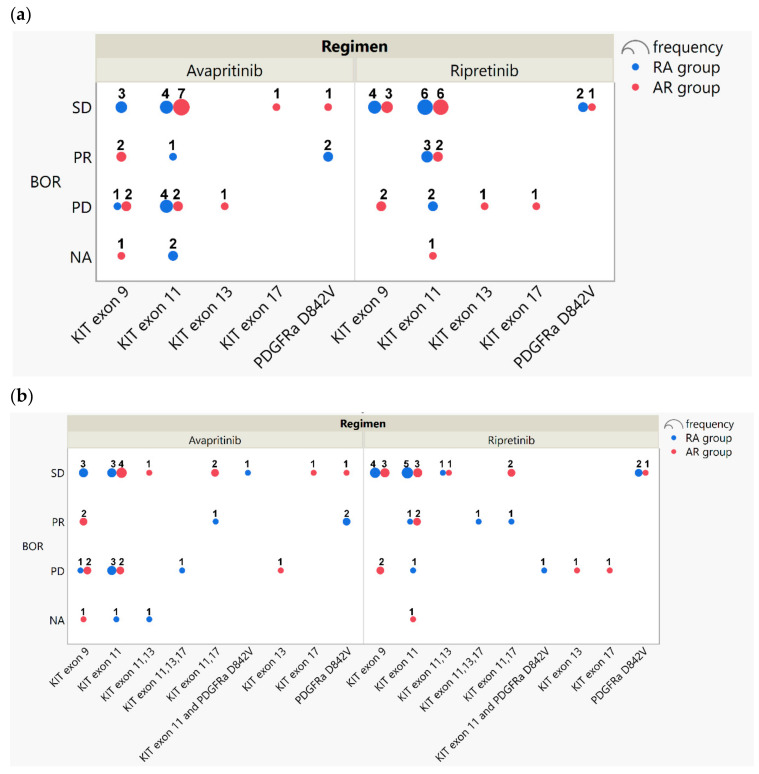
Dot charts showing the frequency of best overall response (BOR) to ripretinib and avapritinib for the RA (blue) and AR (red) treatment sequence groups. (**a**) Best response according to the primary mutation result. (**b**) Best response according to the combination of both primary and secondary mutations. SD, stable disease; PR, partial response; PD, progressive disease; NA, not assessed.

For ripretinib, RRs were 17.6% (3/17) in the RA group and 11.8% (2/17) in the AR group. Response to ripretinib was only observed in patients with the KIT exon 11 primary mutation; among this subset, the RRs were 27.3% (3/11) in the RA group and 22.2% (2/9) in the AR group (Figure 1). Among 13 patients who had a PR and had secondary-mutation data available, both KIT exon 13 and 17 mutations were recorded in the RA group (one patient with exon 11 and 17 mutations and one patient with exon 11, 13, and 17 mutations). Of note, all three patients with the primary PDGFRA D842V mutation had SD as the best response for ripretinib.

For avapritinib as well, RRs were 17.6% (3/17) in the RA group and 11.8% (2/17) in the AR group. In the RA group, PRs were observed in 1 of 11 patients (9.1%) with a primary KIT exon 11 mutation and a secondary exon 17 mutation and both patients with PDGFRA D842V mutations. In the AR group, PRs were seen in two of five patients (40%) with a primary KIT exon 9 mutation.

### 3.3. Long-Term Outcomes

#### 3.3.1. Ripretinib

**Figure 2:**
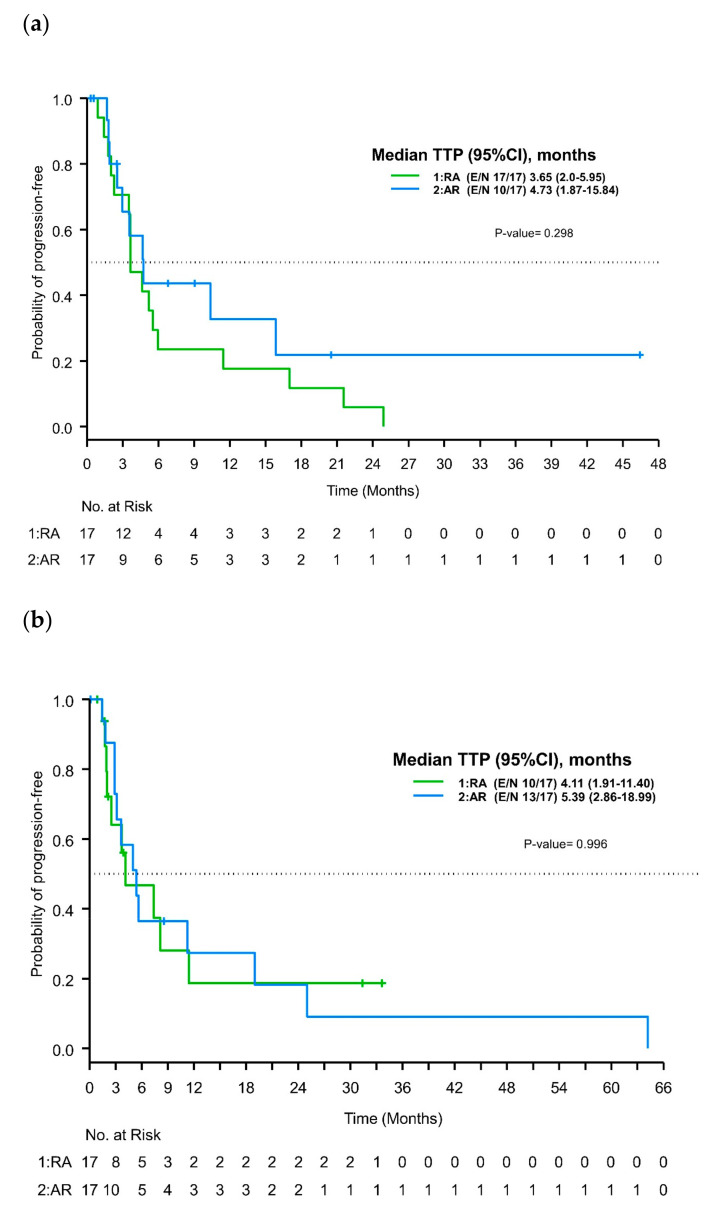
Kaplan–Meier curves for time to disease progression by treatment sequence. (**a**) Time to first progression on ripretinib. (**b**) Time to progression on avapritinib.

The median follow-up time for disease progression in ripretinib treatment records for the whole cohort was 46.4 months (95%CI 9-not reached). At the data cut-point time, 27 of 34 patients had disease progression. Median times to first progression with ripretinib were 3.65 months (95%CI 2–5.95) in the RA group and 4.73 months (95%CI 1.87–15.84) in the AR group. The six-month progression-free rates were 24% (95%CI 0.07–0.45) and 44% (95%CI 0.18–0.67) for the RA and AR groups, respectively, while the twelve-month progression-free rates were 18% (95%CI 0.04–0.38) and 33% (95%CI 0.1–0.59) for the RA and AR groups, respectively. No statistically significant difference was found between these therapy-sequence groups (*p* = 0.30; Figure 2a).

Eleven patients in the RA group (11/17, 64.7%) and four patients in the AR group (4/17, 23.5%) received dose escalation of ripretinib from 150 mg daily to 150 mg twice daily after disease progression. The median follow-up was not reached for the second progression on ripretinib both in the whole cohort (range 2.4 months-NR) and also within each sequence group (2.4 months-NR for RA and 1 month-NR for AR). Nine of eleven patients (81.8%) in the RA group and three of four patients (75%) in the AR group had disease progression. Median TTPs were 4.53 months (95%CI 1.38–15.24) in the RA group and 19.65 months (95%CI 6.01-NR) in the AR group, leading to twelve-month progression-free rates of 23% (95%CI 0.03–0.52) and 67% (95%CI 0.05–0.95) for the RA and AR groups, respectively (*p* = 0.06).

#### 3.3.2. Avapritinib

After the median follow-up of 31.4 months (95%CI 8.5-NR) for avapritinib, 23 of the 34 patients had disease progression. The median TTPs were 4.11 months (95%CI 1.91–11.4) and 5.39 months (95%CI 2.86–18.99) for the RA and AR sequences, respectively. Comparison by log-rank test did not show any statistical significance (*p* = 0.91) (Figure 2b).

#### 3.3.3. Overall Survival Outcome

**Figure 3:**
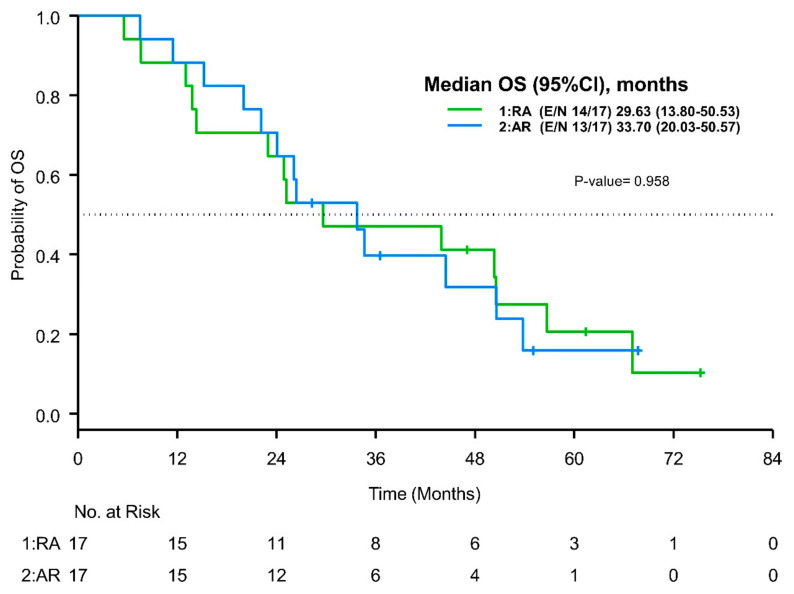
Kaplan–Meier curves for OS by treatment sequence. OS was defined as the time from initiation of the first dose of ripretinib or avapritinib until death or last follow-up.

The median follow-up times for OS were 55.37 months (95%CI 47.03-NR) for the entire cohort: 75.23 months (95%CI 47.03-NR) for the RA group and 55.03 months (95%CI 28.27-NR) for the AR group. The median OS durations were 29.63 months (95%CI 13.8–50.53) for the RA group and 33.7 months (95%CI 20.03–50.57) for the AR group (*p* = 0.96) (Figure 3). The one- and three-year OS rates for the RA group were 75% (95%CI 0.13–0.96) and 25% (95%CI 0.01–0.67), respectively, while the one- and three-year OS rates for the AR group were 100% and 20% (95%CI 0.01–0.58), respectively.

## 4. Discussion

This is the first study to evaluate the efficacy of ripretinib and avapritinib based on the order of their administration. Between MD Anderson’s and OHSU’s GIST clinical programs, we identified a total of 34 patients who had received both drugs (17 patients for each sequence: RA and AR) and retrospectively analyzed response and long-term outcomes, comparing the sequence groups. The primary objective was to elucidate whether there is a benefit of avapritinib in patients who are previously exposed to ripretinib, and vice versa, in the late-line setting of GIST treatment. Furthermore, response in relation to primary and secondary mutations was evaluated.

In our cohort, both TKIs (ripretinib and avapritinib) had comparable RRs and median TTPs, regardless of sequence (RA or AR). Ripretinib demonstrated an RR of 17.6% if given before avapritinib (RA) and 11.8% if given after (AR) in the setting of late-line therapy (median sixth to seventh line). The median TTPs for ripretinib were 3.65 months for the RA sequence and 4.73 months for the AR sequence. These differences between groups were not significant, and these results are in line with the results from the phase 3 INVICTUS study, which had an RR of 11.8% and median PFS of 6.3 months for ripretinib in fourth or later lines of treatment [25].

For avapritinib, we report RRs of 17.6% and 11.8% in the RA and AR groups, respectively. The median TTPs were 4.11 months (95%CI 1.91–11.4) and 5.39 months (95%CI 2.86–18.99) in the RA and AR groups, respectively. These findings were compatible with results from the phase 1 NAVIGATOR study, which revealed an RR of 17% and median PFS of 3.7 months (95%CI 2.8–4.6) in patients with *KIT* or non-D842V *PDGFRA* mutations treated with avapritinib in the fourth- or later-line settings [17].

**Figure 4:**
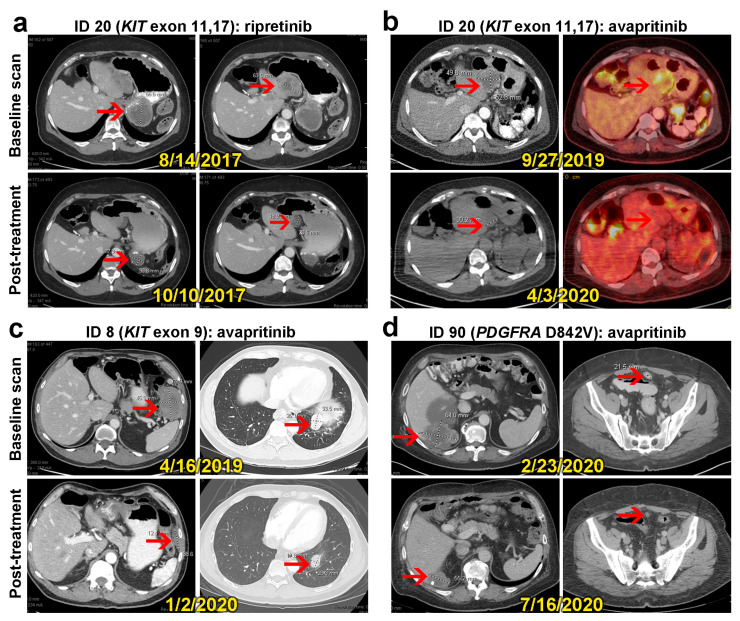
Baseline and post-treatment scans for 3 patients who had treatment response. Red arrows indicate example of target lesions. (**a**,**b**) A patient with metastatic GIST (ID 20) with *KIT* exon 11 and 17 mutations was treated with the RA sequence. The patient had radiographic responses to both ripretinib 150 mg/day and avapritinib 300 mg/day (**a**,**b**). The best response, with 49% volume reduction from baseline scan, was found at 2 months after treatment with ripretinib, before disease progression at the next 12 months (**a**). (**b**) One year after the cessation of ripretinib, the patient began treatment with avapritinib, and treatment response was seen in CT scans of the abdomen (left) and ^18^F-fluorodeoxyglucose positron emission tomography (^18^F-FDG PET) scans (right). The best response (32% volume reduction) was seen in 6 months (**b**). (**c**) A patient with *KIT* exon 9–mutated GIST with lung metastasis (ID 8) who was in the AR group responded to avapritinib. The CT scan with contrast of the chest, abdomen, and pelvis at 8 months after baseline scan showed a 42% decrease in tumor volume. The patient tolerated avapritinib very well and received treatment for 11 months before disease progression. (**d**) A patient with metastatic GIST (ID 90) with a *PDGFRA* D842V primary mutation who was in the RA group showed deep response to avapritinib. However, the disease progressed after 7 months of the treatment, in contrast with another case with the same disease type and mutation who remained responsive to avapritinib after 2 years of treatment.

The current systemic treatment sequence in GIST based on regulatory approval guidelines [26] does not consider subtype of *KIT* mutations; however, emerging prospective data reveal that patient outcomes vary based on activity in distinct *KIT* mutation groups (*KIT* exon 9 vs. 11 and *KIT* exon 13/14 vs. 17/18). In a phase 3 trial exploratory unplanned subgroup analysis of patients with the *KIT* exon 11 and 17/18 mutation or *KIT* AL^pos^ABP^neg^ (based on circulating tumor DNA), ripretinib demonstrated an RR of 40% and median PFS of 14.2 months, while the median PFS for all patients with a primary *KIT* exon 11 mutation was 8.3 months in the second-line setting [27]. In contrast, patients with the *KIT* exon 11 and 13/14 mutation or *KIT* AL^neg^ABP^pos^ had better outcomes when treated with sunitinib. In this population, ripretinib showed an RR of 9.5%, median PFS of 4 months, and median OS of 24.5 months. A previous study of real-world experience with ripretinib in China reported an RR of 25% (2/8) and median PFS of 7.1 months (95%CI 5.1-NR) in patients with the *KIT* exon 11 mutation and an RR of 0% and median PFS of 3.9 months (95%CI 3.7-NR) in patients with the *KIT* exon 9 mutation [28]. In our study, for patients with the *KIT* exon 11 mutation, the RR for ripretinib was 25% (27.3% for RA and 22.2% for AR), and the median TTP on ripretinib was 5.19 months (95%CI 2.53–15.84): 3.65 months (95%CI 1.77–11.43) for RA and 15.84 months (95%CI 1.87-NR) for AR. For patients with the *KIT* exon 9 mutation, there were no objective responses to ripretinib (RR 0%, 0/4 for RA and 0/5 for AR), and the median TTPs were 3.29 months (95%CI 0.89–10.35): 2.81 (95%CI 0.89-NR) for RA and 6.65 (95%CI 1.68-NR) for AR. Due to the limited number of patients in each of the secondary-mutation subgroups, we cannot draw meaningful conclusions about RR and median TTP with ripretinib (two patients with exon 11 and 13 mutations had SD, and three patients with exon 11 and 17 mutations had PR (*n* = 1) or SD (*n* = 2)). Figure 4a shows scans for an RA-group patient with *KIT* exon 11 and 17 mutations who responded well to ripretinib in both local recurrent and metastatic sites.

Recently, a post hoc analysis of the phase 1 NAVIGATOR and phase 1/2 CS3007-101 trials [17] described the efficacy of avapritinib in *KIT*-mutation subgroups. In the *KIT* AL^pos^ABP^neg^ subgroup, avapritinib in all lines of treatment demonstrated an RR of 31.4% and median PFS of 9.1 months, while in the *KIT* exon 9 mutation subgroup, the median PFS durations were 5.6 months in the fourth-line setting (*n* = 14) and 3.7 months in above-fourth-line settings (*n* = 19). In our study, avapritinib use in three patients with *KIT* exon 11 and 17 mutations resulted in PR in one patient in the RA group (Figure 4b) and SD in two patients in the AR group. For the *KIT* exon 9 mutation subgroup (*n* = 9), two patients (22.2%) had PR to avapritinib, both in the AR group (Figure 4c). The median TTP with avapritinib was 3.68 months (95%CI 1.45–8.11): 3.68 months (95%CI 2.5-NR) in the RA group and 3.71 months (95%CI 1.45-NR) in the AR group.

In our cohort, we had three patients with a primary *PDGFRA* D842V mutation (two in the RA group and one in the AR group). The two patients in the RA cohort still obtained PR from avapritinib despite receiving prior treatment with ripretinib, supporting the anti-tumor activity of avapritinib in GIST with *PDGFR* exon 18 mutations even in later lines after ripretinib (Figure 4d), as is well established from prior data [29]. However, the one patient in the AR group with this mutation only had SD with avapritinib. In addition, all three patients had SD as their best response to ripretinib. Of note, one of the RA-group patients with the *PDGFRA* D842V mutation demonstrated an impressive time to first progression on ripretinib of 24.9 months; the other RA-group patient and the AR-group patient had times to first progression of 5.9 months and 4.7 months, respectively.

Although the number of patients with the *PDGFRA* D842V mutation was small, given the long median duration of response (DOR) and median PFS in patients treated with avapritinib in the NAVIGATOR study (DOR, 27.6 months (95%CI 17.6-NR); PFS, 34 months (95%CI 22.9-NR)) [30], the outcome of these patients could possibly affect the endpoints for our entire cohort. Hence, we also analyzed long-term outcomes of our cohort with the exclusion of *PDGFRA* D842V patients: the median times to first progression for ripretinib were 3.65 months (95%CI 1.77–5.19) in the RA group and 4.67 months (95%CI 1.87-NR) in the AR group (*p* = 0.19). The median TTPs for avapritinib were 3.68 months (95%CI 1.91–11.4) in the RA group and 4.96 months (95%CI 2.86–11.24) in the AR group (*p* = 0.73). The median OS durations from the beginning of the RA and AR treatment sequences were also not significantly different (25.17 months (95%CI 13–50.33) for RA and 30.03 months (95%CI 20.03–50.57) for AR; *p* = 0.80).

The numerical differences in TTP for ripretinib and avapritinib and OS favoring the AR group did not show significance after the statistical analysis, suggesting that they might be by chance, except for the TTP for ripretinib, which in the Kaplan–Meier curve showed a longer tail than the compared arm. We believe that the curve is showing a few patients who received more prolonged benefit from ripretinib than the others. This could be the result of the two patients who had very good response and received local treatment during ripretinib treatment.

From our analysis of 11 patients in the RA group (11/17, 64.7%) and 4 patients in the AR group (4/17, 23.5%) who received dose escalation of ripretinib after disease progression, there seemed to be benefit in increasing the dose; the additional median TTP was 6.37 months (95%CI 2.96–16.07): 4.53 months (95%CI 1.38–15.24) for RA and 19.65 months (95%CI 6.01-NR) for AR. Since the number of patients with dose escalation in the AR group was limited, a conclusion on whether the AR group had more benefit from increasing the dose cannot be made.

Despite showing comparable efficacy of both ripretinib and avapritinib, the AR group had double the number of treatment discontinuations due to the toxicity of both drugs (5/17 for avapritinib and 4/17 for ripretinib). There is no clear reason as to why there was a higher percentage of avapritinib discontinuation due to toxicity for AR (5/17, 29.4% vs. 2/17, 11.8% for RA). Based on a previous clinical trial in the fourth- or later-line setting, avapritinib had a fair tolerability profile, with 51% of the patients (*n* = 154) experiencing grade ≥ 3 overall treatment-related adverse events, 2.6% experiencing grade ≥ 3 cognitive effects, and 20% having adverse events leading to study discontinuation in the 300 mg daily dose group [31]. In this study, the most common adverse event leading to discontinuation for avapritinib was cognitive effects requiring hospitalization, which occurred in four patients: one for RA and three for AR (Table S3, Supplementary Data). Albeit being reversible symptoms, none of the patients were willing to continue the treatment drug. The previous clinical trials reported that overall incidence of cognitive effects was numerically higher in the patients who initiated avapritinib at a higher dose [31,32]. Additionally, a post hoc analysis of data from the NAVIGATOR study found that the cognitive effects occurred more frequently in elderly patients (defined as ≥65 years) than in younger patients (*p* = 0.018) but were not associated with cumulative dose, race, gender, baseline ECOG performance status, nor number/duration of prior TKIs used [33]. In our study, the AR group’s median age at the time of treatment initiation was 57.4 years (range 39.0–78.0), which was comparable to that of the RA group, 56.2 years (range 27.8–75.3) (Table 2). The patients in the AR group who experienced severe cognitive effects were 48.8, 72.2, and 68.9 years of age. With our limited number of events, we cannot conclude whether any factor might relate to the adverse effects.

The adverse effect profile of ripretinib is different from that of avapritinib but also found more frequently with increased dose [30]. In the phase 1 study that included dose escalation and expansion (*n* = 179), 13% of the patients (*n* = 24) had to discontinue treatment due to treatment-emergent adverse events, with increased lipase level, anemia, hypertension, and abdominal pain being the more common (>5%) of the grade 3/4 adverse events found [34,35]. In the phase 3 INTRIGUE study, comparing ripretinib 150 mg to sunitinib in the second-line setting, only 3.6% of the patients (*n* = 223) experienced treatment-emergent adverse events, leading to study treatment discontinuation, while 41.3% of those patients had any grade 3/4 adverse events [36]. The most common were alopecia, fatigue, and myalgia. In our study, we reported adverse events leading to treatment discontinuation in 11.8% (2/17) for RA and 23.5% (4/17) for AR. The three MD Anderson patients who discontinued ripretinib owing to toxicity were the same ones who could not tolerate avapritinib and had not obtained any tumor shrinkage while on avapritinib. One of the three patients showed significant performance status decline during avapritinib treatment. With the limited number of events, together with the decision to stop treatment being made in consideration of other factors as opposed to in the trial setting, we cannot conclude that the sequence affected the tolerability of the drug treatments.

After the emergence of the use of TKIs targeting *KIT/PDGFRA* [37], the main systemic therapy development in GIST has targeted ABP and AL mutations, which are found to be present after treatment with imatinib. As compared to other more common cancers such as lung and breast cancer, there is still a lack of revolutionary improvement that might be able to lead to long-term disease control. As we observed, even targeting the same mutation produced differences in the benefit each patient received. We believe that other mechanisms also may play a role in the later-line disease.

## 5. Conclusions

In sum, we found that both ripretinib and avapritinib are efficacious in later lines of treatment, and there is no evidence of reduced efficacy of one agent following treatment with the other. Comparable RRs, median TTPs, and median OS durations were seen with each of the drugs, regardless of sequence.

Although our study is a retrospective study, we are the first to report outcomes in patients who were treated with both ripretinib and avapritinib. Moreover, our population was well balanced, and the results were in line with the previous report from a larger retrospective study and randomized controlled studies. Our result emphasizes the impact of mutational analysis in relation to the response of ripretinib and avapritinib, especially those with the exon 9 and AL *KIT* mutation. In current clinical practice, sending new tissue biopsy or liquid biopsy for secondary mutation testing might be inconvenient and without benefit in most patients. However, mutation testing would be more crucial in situations in which good response is required in a limited timeframe, such as when locally advanced disease is compromising other organs or surgery is intended, or in the case of disease that is inconceivably resistant to the treatments. Also, the same concept of choosing a drug according to its sensitivity profile might be considered in an earlier line of treatment, which may be more likely to have an impact on patient survival. Further studies or analyses are encouraged to move forward to a more personalized therapy strategy.

## Figures and Tables

**Table 1:** Baseline characteristics.

Characteristics *n* (%)		Total (*n* = 34)	RA Group (*n* = 17)	AR Group (*n* = 17)	*p*-Value
Age at diagnosis, median (range), years		48.3 (20.3–76.3)	49.5 (20.3–67.2)	46.0 (34.5–76.3)	0.45
Sex	Male	22 (64.7)	10 (58.8)	12 (70.6)	0.72
	Female	12 (35.3)	7 (41.2)	5 (29.4)	
Primary site	Stomach	10 (29.4)	6 (35.3)	4 (23.5)	0.41
	Small bowel	17 (50.0)	9 (52.9)	8 (47.1)	
	Rectum	3 (8.8)	0 (0)	3 (17.6)	
	Others	4 (11.8)	2 (11.8)	2 (11.8)	
Histologic subtype	Spindle cell	23 (67.6)	9 (52.9)	14 (82.4)	0.24
	Epithelioid	1 (2.9)	1 (5.9)	0 (0)	
	Mixed type	5 (14.7)	4 (23.5)	1 (5.9)	
	Not reported	5 (14.7)	3 (17.6)	2 (11.8)	
Primary mutation	*KIT* exon 9	9 (26.5)	4 (23.5)	5 (29.4)	0.82
	*KIT* exon 11	20 (58.8)	11 (64.7)	9 (52.9)	
	*KIT* exon 13	1 (2.9)	0 (0)	1 (5.9)	
	*KIT* exon 17	1 (2.9)	0 (0)	1 (5.9)	
	*PDGFRA* D842V	3 (8.8)	2 (11.8)	1 (5.9)	
Secondary mutation/additional mutation detected	*KIT* exon 13	2 (5.9)	1 (5.9)	1 (5.9)	1.0
	*KIT* exon 17	3 (8.8)	1 (5.9)	2 (11.8)	
	*KIT* exons 13 and 17	1 (2.9)	1 (5.9)	0 (0)	
	*PDGFRA* D842V	1 (2.9)	1 (5.9)	0 (0)	
	Not detected	6 (17.6)	5 (29.4)	1 (5.9)	
	Not tested or unknown	21 (61.8)	8 (47.1)	13 (76.5)	
Disease status and treatment at primary presentation	Localized	14 (41.2)	5 (29.4)	9 (52.9)	0.32
	- Surgery	14	5	9	
	- Surgery + systemic Rx (neoadjuvant/adjuvant)	2	1	1	
	Locally advanced	11 (32.4)	6 (35.3)	5 (29.4)	
	- Surgery	2	1	1	
	- Surgery + systemic Rx (neoadjuvant/adjuvant)	8	5	3	
	- Systemic Rx alone	1	0	1	
	Metastatic	9 (26.5)	6 (35.3)	3 (17.6)	
	- Surgery (primary and metastatectomy)	1	1	0	
	- Surgery + systemic Rx	3	3	0	
	- Systemic Rx alone	5	2	3	

RA, ripretinib–avapritinib sequence; AR, avapritinib–ripretinib sequence; *KIT*, KIT proto-oncogene, receptor tyrosine kinase; *PDGFRA*, platelet-derived growth factor receptor alpha; Rx, therapy.

**Table 2:** Details of ripretinib and avapritinib treatment in the RA and AR groups.

Treatment	RA (*n* = 17)		AR (*n* = 17)	
	Ripretinib *	Avapritinib	Avapritinib	Ripretinib *
Age at the start of treatment sequence, median (range), years	56.2 (27.8–75.3)		57.4 (39.0–78.0)	
Lines of Rx, median (range)	5 (3–8)	6 (4–10)	5 (2–9)	6 (4–10)
Local treatment during systemic Rx, *n* (%)	1 (5.9)	1 (5.9)	1 (5.9)	3 (17.6)
Duration of treatment with either drug, median (range), days	348.0 (58–1351)	154.5 (26–1643)	110.0 (3–1954)	214.0 (9–1478)
- Reason for treatment discontinuation, *n* (%)				
- Radiographic progression	15 (88.2)	9 (52.9)	12 (70.6)	9 (52.9)
- Clinical progression	0	2 (11.8)	0	1 (5.9)
- Toxicity **	2 (11.8)	2 (11.8)	5 (29.4)	4 (23.5)
- Death, unknown cause	0	2 (11.8)	0	1 (5.9)
- Ongoing treatment	0	2 (11.8)	0	2 (11.8)

* Eleven patients in the RA group and four patients in the AR group received ripretinib dose escalation to 300 mg/day after progression on a 150 mg/day dose. ** Details of toxic effects leading to treatment termination are presented in Table S3 (Supplementary Data).

## Data Availability

The data that support the findings of this study are available on request from the corresponding author (N.S.).

## Supplementary Materials

The following supporting information can be downloaded at: https://www.mdpi.com/article/10.3390/cancers16050904/s1, Table S1. Frequency of mutations detected by primary tumor site; Table S2. Details of surgical operations in the primary treatment setting for each treatment sequence group; Table S3. Patients’ toxic reactions that led to treatment discontinuation.
