# Supplementary material for: Outcomes of Late-Line Systemic Treatment in GIST: Does Sequence Matter?
Source: Cancers (Basel). 2024 Feb 23;16(5):904. doi: 10.3390/cancers16050904 (PMC10931337; doi:10.3390/cancers16050904)
Supplement: Supplementary file 1 [file cancers-16-00904-s001.zip › cancers-2825776-supplementary.pdf]

Supplementary Data

Table S1. Frequency of mutations detected by primary tumor site

**Mutation**		**Primary tumor site n, (%)**				
		**Stomach (n=10)**	**Small bowel (n=17)**	**Rectum (n=3)**	**Intra-abdomen, NOS (n=3)**	**Omentum (n=1)**
Primary mutation	*KIT* exon 9 (n=9)	0 (0)	9 (52.9)	0 (0)	0 (0)	0 (0)
	KIT exon 11 (n=20)	10 (100)	7 (41.2)	2 (66.7)	0 (0)	1 (100)
	KIT exon 13 (n=1)	0 (0)	0 (0)	1 (33.3)	0 (0)	0 (0)
	KIT exon 17 (n=1)	0 (0)	1 (5.9)	0 (0)	0 (0)	0 (0)
	PDGFRA D842V (n=3)	0 (0)	0 (0)	0 (0)	3 (100)	0 (0)
Overall mutation	*KIT* exon 9 (n=9)	0 (0)	9 (52.9)	0 (0)	0 (0)	0 (0)
	KIT exon 11 (n=13)	7 (70.0)	5 (29.4)	0 (0)	0 (0)	1 (100)
	KIT exon 11, 13 (n=2)	1 (10.0)	0 (0)	1 (33.3)	0 (0)	0 (0)
	KIT exon 11, 17 (n=3)	1 (10.0)	1 (5.9)	1 (33.3)	0 (0)	0 (0)
	KIT exon 11, 13, 17 (n=1)	1 (10.0)	0 (0)	0 (0)	0 (0)	0 (0)
	KIT exon 11 + *PDGFRA* D842V (n=1)	0 (0)	1 (5.9)	0 (0)	0 (0)	0 (0)
	KIT exon 13 (n=1)	0 (0)	0 (0)	1 (33.3)	0 (0)	0 (0)
	KIT exon 17 (n=1)	0 (0)	1 (5.9)	0 (0)	0 (0)	0 (0)
	PDGFRA D842V (n=3)	0 (0)	0 (0)	0 (0)	3 (100)	0 (0)

NOS, not otherwise specified (no specific primary site found)

Table S2. Details of surgical operations in the primary treatment setting for each treatment sequence group

Disease status at diagnosis	Surgery name	RA, n	AR, n
Localized disease (n=14)	Small bowel resection	2	5
	Abdominal tumor mass excision	2	0
	Partial gastrectomy	1	3
	Low anterior resection	0	1
Locally advanced disease (n=10)	Small bowel resection	2	1
	Small bowel resection with segmental hepatectomy	1	0
	Partial gastrectomy	1	0
	Partial gastrectomy with distal pancreatectomy, splenectomy and appendectomy	1	0
	Abdominal tumor mass excision	1	1
	Hysterectomy with BSO, staging and open laparotomy, small bowel resection, partial omentectomy, pelvic LN dissection, peritoneal sampling	0	1
	Radical resection of perirectal GIST, including proctectomy, distal sigmoid colon resection, partial prostatectomy and resection of right vas deferens in seminal vesicle	0	1
Metastatic disease (n=4)	Small bowel mass resection	1	0
	Small bowel resection with omental nodule excision	1	0
	Partial gastrectomy with right hepatic lobectomy	1	0
	Resection of gastric wall mass and peritoneal implant	1	0

BSO, bilateral salpingo-oophorectomy; LN, lymph node; GIST, gastrointestinal stromal tumor

Table S3. Patients' toxic reactions that led to treatment discontinuation.

Treatment group	Patient ID	Drug	
		**Ripretinib**	**Avapritinib**
**RA**	ID 20	Cardiotoxicity, decreased ejection fraction	Diarrhea, declined mental status requiring hospitalization
	ID 21	Increased lipase level, possible pancreatitis	
	ID 54		Septic shock, disseminated intravascular coagulation, death
**AR**	ID 18	Tremors, palpitations	Neurological symptoms requiring hospitalization: aphasia, hallucination, weakness (subsequently resolved)
	ID 64	Severe fatigue	Hyperbilirubinemia (Gilbert disease; hyperbilirubinemia was asymptomatic, but had to stop drug per protocol)
	ID 89	Worsening of performance status	Status deterioration, small bowel obstruction
	ID 102		Neurologic symptoms, headache, auditory hallucination, nausea/vomiting (MRI of brain did not reveal etiology)
	ID 104		Altered sensorium and memory deficits
	ID P3	Nausea/vomiting	

MRI, magnetic resonance imaging
